# Comparison of physician referral and insurance claims data-based risk prediction as approaches to identify patients for care management in primary care: an observational study

**DOI:** 10.1186/1471-2296-14-157

**Published:** 2013-10-20

**Authors:** Tobias Freund, Matthias Gondan, Justine Rochon, Frank Peters-Klimm, Stephen Campbell, Michel Wensing, Joachim Szecsenyi

**Affiliations:** 1 Department of General Practice and Health Services Research, University Hospital Heidelberg, Vossstrasse 2, D-69115 Heidelberg, Germany; 2 Institute of Medical Biometry and Informatics, University of Heidelberg, Heidelberg, Germany; 3 Centre for Primary Care, Institute for Population Health, University of Manchester, Manchester, UK; 4 Centre for Quality of Care Research, Radboud University Nijmegen Medical Centre, Nijmegen, Netherlands

**Keywords:** Decision support techniques, Case finding, Case management, Disease management, Primary care, Multimorbidity, Avoidable hospitalization, Prediction

## Abstract

**Background:**

Primary care-based care management (CM) could reduce hospital admissions in high-risk patients. Identification of patients most likely to benefit is needed as resources for CM are limited. This study aimed to compare hospitalization and mortality rates of patients identified for CM either by treating primary care physicians (PCPs) or predictive modelling software for hospitalization risk (PM).

**Methods:**

In 2009, a cohort of 6,026 beneficiaries of a German statutory health insurance served as a sample for patient identification for CM by PCPs or commercial PM (CSSG 0.8, Verisk Health). The resulting samples were compared regarding hospitalization and mortality rates in 2010 and in the two year period before patient selection. No CM-intervention was delivered until the end of 2010 and PCPs were blinded for the assessment of hospitalization rates.

**Results:**

In 2010, hospitalization rates of PM-identified patients were 80% higher compared to PCP-identified patients. Mortality rates were also 8% higher in PM-identified patients if compared to PCP-identified patients (10% vs. 2%). The hospitalization rate of patients independently identified by both PM and PCPs was numerically between PM- and PCP-identified patients. Time trend between 2007 and 2010 showed decreasing hospitalization rates in PM-identified patients (−15% per year) compared to increasing rates in PCP-identified patients (+34% per year).

**Conclusions:**

PM identified patients with higher hospitalization and mortality rates compared to PCP-referred patients. But the latter showed increasing hospitalization rates over time thereby suggesting that PCPs may be able to predict future deterioration in patients with relatively good current health status. These patients may most likely benefit from preventive services like CM.

## Background

An increasing number of patients with complex care needs resulting from multiple chronic conditions challenge health care systems worldwide [1]. Care management (CM) programs have been developed to meet these needs and are often delivered by multi-professional teams. CM commonly consists of four key elements: i) assessment of patients’ needs and resources, ii) individualized goal setting and action planning, iii) specific actions to achieve these goals (e.g. self-management education, medication counselling), and iv) frequent monitoring of symptoms and goal attainment, often delivered by phone [2]. CM programs show promising results particularly if focused on patients at high risk of future deterioration [3,4]. Therefore, identification of patients likely to benefit – commonly called ‘case finding’ - appears to be essential. Beside physician referral health insurance companies and physician groups increasingly use computerized tools to predict individual patient outcomes so that CM could be targeted to patients at high risk of future health care utilization [5]. These models often rely on insurance claims data including inpatient and outpatient diagnosis, medication and prior healthcare utilization. As data from electronic medical records are increasingly available, information technology will further use these ‘big data’ to predict future outcomes in order to inform the identification of patients deemed to be most likely to benefit from CM [5].

Previous research has shown that, compared to patients identified by a predictive modelling software (PM), primary care physicians (PCPs) refer patients with lower *predicted* healthcare utilization but higher ‘care sensitivity’, that is, willingness and ability to participate in a program which is able to address their health needs [6,7]. However, little is known about the ‘predictive value’ of physician referral regarding future healthcare utilization and ‘care sensitivity’ if compared to PM. Therefore, this study aimed to compare the natural course of hospitalization and mortality rates (as a partial proxy for care sensitivity) in patients identified as potential participants of a CM either by treating PCPs or PM.

## Methods

In the fall of 2009, a comprehensive sample of all beneficiaries of the General Regional Health Fund (*Allgemeine Ortskrankenkasse* [AOK]) from 10 primary care practices in south-western Germany were screened for potential participants of a CM intervention comparing patient identification by PCP or PM. Further details of the selection process and patients’ characteristics at baseline have been published previously [6]. The 10 primary care practices (5 single-handed practices and 5 group practices) were recruited from rural areas (3 practices) and (sub-)urban areas (7 practices). We obtained de-identified insurance claims data including medical and pharmacy claims from January 2007 to December 2010 from AOK beneficiaries of all ages. This study was part of a series of studies to develop a CM program for high-risk patients in primary care [8]. The University Hospital Heidelberg Institutional Review Board approved the study.

### Predictive modelling software

For PM-based case finding, the AOK used the commercial software package *Case Smart Suite Germany* (Verisk Health, Munich, Germany) [9]. This program is an extension of diagnostic cost group-PM, which has been applied previously in comparative case finding studies [10]. Information from the past 2 years (2007–2008) served as inputs for PM, including all *International Statistical Classification of Diseases,* (10th Revision, German modi-fication, *ICD-10-GM*) diagnostic codes assigned in outpatient and inpatient settings, prior costs, hospital admissions, and demographic data. Based on multivariate logistic regression analysis, the software computes the likelihood of at least one hospitalization (LOH) for each individual within the next 12 months (2010). In our study, patients with a LOH score above the 90th percentile of the sample were classified as ‘high-risk’ and identified as potential participants of CM without any restrictions of the number of patients selected per practice. Due to the design of the planned CM intervention we focused on patients with at least one of the following *ICD-10-GM* index conditions [11]: type 2 diabetes mellitus (codes E11-E14), chronic obstructive pulmonary disease (J43-J44), asthma (J45), chronic heart failure (I11.0, I13.0, I13.2, I25.5, I50), and late-life depression (F32-F33 [>60 years]). Patients with dementia (F00-F03), dialysis (Z49, Z99.2), or active cancer disease (C00-C97) were excluded.

### Physician referral

Fourteen PCPs from the 10 participating primary care practices were asked to screen a list of all AOK beneficiaries in their practice and to identify up to 30 patients for future participation in a CM program aimed at reducing avoidable hospitalizations. PCPs were informed about the aims and intervention elements of the planned CM intervention but no explicit selection criteria were given in addition to the inclusion and exclusion criteria above. PCPs were blinded to results of PM until they submitted their final list of selected patients.

### Intervention and outcomes

In the year following patient selection *no* intervention beside best generally available primary care took place in either of the groups (the CM intervention started by the end of 2010). Data on hospital admissions and mortality were obtained from insurance claims thereby minimizing reporting bias. PCPs were blinded for the assessment of hospitalization rates in 2010.

### Statistical analysis

Statistical analyses were carried out with R version 2.15.1 [12]. Continuous data were summarized using medians and interquartile ranges (1st and 3rd quartiles) and categorical data using frequency counts and percentages. Because of the hierarchical structure of the data, multilevel analysis was applied that took into account the dependence between patient outcomes (level 1) within primary care practices (level 2). The number of hospitalizations was analysed by a multilevel Poisson regression model [13], with ‘Group’ (not selected, PM, PCP, both) as a categorical predictor, and random intercepts accounting for overdispersion due to differences across practices. In longitudinal data analyses, an additional covariate ‘Year’ was used in the regression model, and an additional random intercept accounted for the correlated outcomes within patients. Results are reported as risk ratios (RR) with 95% Wald-type confidence intervals (CI). Some of the patients died within 2010. Since the exact date of death was not included in the data we did not adjust for reduced exposition in these patients. However, in a sensitivity analysis, we included an offset variable in the Poisson regression for patients who died in 2010, thereby assuming that these patients survived 6 months (until 30 June 2010). Results of this sensitivity analysis are reported when they differ from the main analysis.

Mortality rates were investigated using a multilevel binomial logistic regression model, with ‘Group’ as a categorical predictor and random intercepts for practices. Results are reported as odds ratios (OR) with 95% CIs. In line with the exploratory nature of this study, the significance level was set to 5% (two-sided), and we performed neither adjustment for multiple testing nor imputation of missing values.

## Results

Complete data on hospitalizations between 2007 and 2010 were available for 5,865 AOK beneficiaries (97%). On average, PCPs screened 464 beneficiaries per practice and identified 20 patients as potential future participants in CM. On average, PM identified 28 patients per practice. Characteristics of the selected patients are shown in Table 1.

**Table 1:** **Characteristics of the patient cohort (*n*** **= 5865)**

**Selection**	**No**	**PCP**	**PM**	**PM + PCP**
*n*	5351	203	281	30
Female, *n* (%)	2247 (42)	95 (47)	112 (40)	14 (47)
Age, median (IQR)	56 (40–71)	70 (61–75)	78 (70–83)	75 (70–81)
≥ 1 hospital admission				
2007, *n* (%)	892 (17)	33 (16)	196 (70)	18 (60)
2008	855 (16)	45 (22)	185 (66)	16 (53)
2009	964 (18)	55 (27)	131 (47)	10 (33)
2010	997 (19)	53 (26)	139 (49)	13 (43)

IQR: Interquartile range (1^st^– ^3rd^ Quartile).

**Figure 1:**
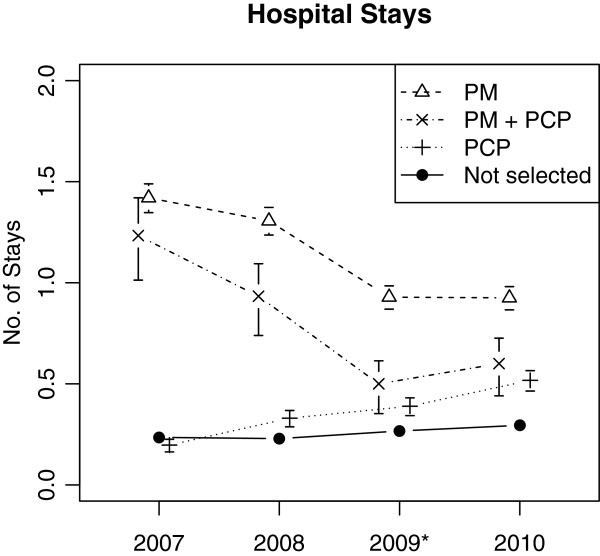
**Hospitalizations in patients selected by physician, software or both.** Mean number of hospitalizations per patient per year (Poisson rate estimates with standard errors) for patients independently identified as potential participants of a care management program in 2009 (marked by asterisk) by primary care physician (PCP), predictive modelling (PM) or both (PM + PCP).

In non-selected patients (*n* = 5,351) hospitalization rates per patient per year steadily increased by 9% annually (RR = 1.09, 95% CI from 1.07 to 1.11, *p* < .001) (Figure 1). Patients identified by PCPs showed a higher increase of hospitalization rates over time (by 34% per year, RR = 1.34, CI 1.21 to 1.48, *p* < .001) whereas hospitalization rates of patients identified by PM decreased by 15% each year (RR = 0.85, CI 0.80 to 0.90, *p* < .001).

In 2010, the year following patient selection, PCP-identified patients had a 76% increased risk of hospitalization (RR: 1.76, CI 1.32 to 2.33, *p* < .001) compared to non-selected patients. Those identified by PM had even higher hospitalization rates (compared to non-selected: RR = 3.14, CI 2.60 to 3.79, *p* < .001; compared to PCP-identified patients: RR = 1.80, CI 1.28 to 2.53, *p* < .001). The hospitalization rate of patients independently identified by both PM and PCPs was numerically between PM- and PCP-identified patients (compared to non-selected: RR = 2.04, CI 1.05 to 3.95, *p* = .036) [Here, the sensitivity analysis with adjusted exposition time for patients that died in 2010 yielded a slightly different result: RR = 2.02, CI 1.00 to 4.06, p = .050 n.s.].

The positive predictive value of at least one hospitalization in 2010 was 28% (PCP-selection), 49% (PM-selection) and 43% (PCP + PM selection) with sensitivity values of 5% (PCP-selection), 12% (PM-selection) and 1% (PCP + PM selection).

A total number of 139 patients died in 2010: 107 non-selected (2%), 4 PCP-identified (2%), 28 PM-identified (10%), 0 PCP + PM-identified (0%). Thus, PM-identified patients had a significantly higher mortality compared to non-selected patients (OR = 5.47, CI 3.56 to 8.42, *p* < .001), whereas no statistically significant association with mortality was observed for identification by PCPs (OR = 0.97; CI 0.36 to 2.62, *p* = .946).

## Discussion

In the year after identification, PM-identified patients had higher hospitalisation and mortality rates than PCP-identified patients. However, without receiving any planned intervention, PCP-identified patients showed increasing hospitalization rates over time. In contrast, hospitalization rates of patients identified by PM were decreasing over time. Compared to referral by PCPs, PM identified patients with higher hospitalization rates at each time point under consideration. However, PM-identified patients also showed significantly higher short-term mortality.

Given the resources needed to run CM programs in primary care (e.g. time efforts, personnel costs), CM programs are thought to be most effective in patients with high risk of future healthcare utilization [3]. However, routine use of PM as a case finding tool for CM has to pay off by its superior accuracy in predicting outcomes when compared to PCP referral given the resources needed for data supply and software licensing. In general, PM shows limited performance on the prediction of future (re-)hospitalizations [14]. However, our data support the hypothesis that PM is superior in predicting future hospitalization compared to PCPs if comparing *absolute* risk of hospital admissions per patient alone. If the trend of hospitalizations over time is compared, PCPs but not PM identified patients with increasing hospital admissions per year. One possible explanation for this phenomenon may be the fact, that PCPs are particularly in knowledge of patients’ medical and social needs commonly linked to ‘potentially avoidable hospitalizations’ [15]. PCP referral is the default access to CM programs in most health systems. It may be useful if patients are not insured (or insured too short for the data input needed to run a PM) or in case PM is not available (e.g. small- to middle-sized primary care practices).

Furthermore, the process of patient identification by PM *and* PCP requires consideration. Patient identification by PM-systems, by design, mainly carries forward the health status observed in the past and tries to yield a precise likelihood of future hospitalization. Despite regression to the mean (see trends in Figure 1), PM seems to work well in predicting hospitalizations. However, as the increased mortality in PM-selected patients may suggest, PM is less able to take into account the ability of patients to participate in and benefit from CM programs [6,16]. As we learned from a qualitative analysis, PCPs refer those patients who may have had a good health status in the past but are at risk of future deterioration and likely to be willing and able to participate in CM programs whereas PM tends to identify a significant proportion of patients who are not (longer) amenable to care due to extreme high morbidity burden or fatal clinical status [7]. Furthermore, PCPs tend to select patients who participated in preventive chronic care services which may be seen as an indicator of their willingness to participate in CM [6]. Potentially avoidable hospitalizations have complex causes including social determinants, patient behavior and causes in the healthcare system (e.g. lack of ambulatory resources) but not all of them could be addressed by CM [17,18]. Whereas treating PCPs are familiar with these causes in most individuals, which may influence their rating of patients’ likelihood to benefit from CM, to date, PM is blinded for a number of relevant independent variables contributing to hospitalizations as well as to patients’ likelihood to benefit from preventive measures like CM [15]. Therefore, a combined approach of PM selection and PCP screening appears to be most promising as high risk patients with low care sensitivity who are identified by PM may be excluded by PCPs who are aware of the complex contextual factors contributing to a patients’ risk of future hospitalization. However, the proportion of patients identified by both approaches appears to be very low. Future prospective intervention studies will have to determine if narrowly focusing CM on high-risk but care sensitive patients has enough marginal benefit to offset the loss of a large proportion of patients not included by either of the approaches.

### Limitations

One limitation of this research was the small group of patients independently identified by both PM and PCPs. For example, with only about 30 patients selected by both PM and PCPs, and 200 patients selected by PCPs, and the conventional *α* = 5% two-sided, differences in mortality of more than 20% are needed to obtain 90% statistical power in the comparison. Moreover, we were not able to use more than one PM software which was applied by the German statutory health insurance. Nevertheless, the PM software used in the present study is widely used as a case finding software and showed similar characteristics to those commonly used in other countries [9,10,14].

## Conclusions

In conclusion, our findings support identifying high risk patients for CM programs by using PM software. However, as treating PCPs are able to select potential participants based on comprehensive knowledge about a patients’ clinical, social and behavioural risk, their rating is an essential complement to PM-based case finding highlighting patients’ overall care sensitivity.

## Abbreviations

PCP: Primary care physician; PM: Predictive modelling; CM: Care management; IQR: Interquartile range (1st– 3rd Quartile).

## Competing interests

The authors declare that they have no competing interest.

## Authors’ contributions

TF designed the study and drafted the manuscript. MG and JR performed the statistical analyses and helped to draft the manuscript. FPK, SC, MW and JS participated in the design of the study and helped to draft the manuscript. All authors read and approved the final manuscript.

## Pre-publication history

The pre-publication history for this paper can be accessed here:

http://www.biomedcentral.com/1471-2296/14/157/prepub
